# Identification of porcine fast/slow myogenic exosomes and their regulatory effects on lipid accumulation in intramuscular adipocytes

**DOI:** 10.1186/s40104-024-01029-0

**Published:** 2024-06-02

**Authors:** Tiantian Zhao, Tingting Tian, He Yu, Chaoyue Cao, Ziyi Zhang, Zhaozhao He, Zeqiang Ma, Rui Cai, Fengna Li, Weijun Pang

**Affiliations:** 1 Laboratory of Animal Fat Deposition & Muscle Development, College of Animal Science and Technology, Northwest A&F University, Yangling, 712100 Shaanxi China; 2 Laboratory of Animal Nutritional Physiology and Metabolic Process, Key Laboratory of Agro-ecological Processes in Subtropical Region, Institute of Subtropical Agriculture, Chinese Academy of Sciences, Changsha, 410125 Hunan China

**Keywords:** Adipogenesis, Exosome, Extensor digitorum longus, Intramuscular adipocyte, Muscle-fat tissue interaction, Pig, Soleus

## Abstract

**Background:**

Pork quality is affected by the type of muscle fibers, which is closely related to meat color, tenderness and juiciness. Exosomes are tiny vesicles with a diameter of approximately 30–150 nm that are secreted by cells and taken up by recipient cells to mediate communication. Exosome-mediated muscle-fat tissue crosstalk is a newly discovered mechanism that may have an important effect on intramuscular fat deposition and with that on meat quality. Various of adipose tissue-derived exosomes have been discovered and identified, but the identification and function of muscle exosomes, especially porcine fast/slow myotube exosomes, remain unclear. Here, we first isolated and identified exosomes secreted from porcine extensor digitorum longus (EDL) and soleus (SOL), which represent fast and slow muscle, respectively, and further explored their effects on lipid accumulation in longissimus dorsi adipocytes.

**Results:**

Porcine SOL-derived exosomes (SOL-EXO) and EDL-derived exosomes (EDL-EXO) were first identified and their average particle sizes were approximately 84 nm with double-membrane disc- shapes as observed via transmission electron microscopy and scanning electron microscopy. Moreover, the intramuscular fat content of the SOL was greater than that of the EDL at 180 days of age, because SOL intramuscular adipocytes had a stronger lipid-accumulating capacity than those of the EDL. Raman spectral analysis revealed that SOL-EXO protein content was much greater than that of EDL-EXO. Proteomic sequencing identified 72 proteins that were significantly differentially expressed between SOL-EXO and EDL-EXO, 31 of which were downregulated and 41 of which were upregulated in SOL-EXO.

**Conclusions:**

Our findings suggest that muscle-fat tissue interactions occur partly via SOL-EXO promoting adipogenic activity of intramuscular adipocytes.

**Supplementary Information:**

The online version contains supplementary material available at 10.1186/s40104-024-01029-0.

## Background

Pork is the most commonly consumed meat in China due to its delicacy, high yield and low price [1, 2]. Adipose tissue is essential for livestock and is involved in the composition of living organisms [3], but can also affect pork quality. Adipose tissue can often be subdivided into three separate compartments, namely, the intramuscular fat (IMF), subcutaneous fat, and visceral fat, of which the IMF content is an important factor affecting meat quality [4]. An appropriate amount of IMF (marbling) in pork results in a good meat quality, and an IMF content of 2.5%–3% in pork is generally believed to be optimal [5]. However, limited information is available on the IMF quality traits of skeletal muscles with different anatomical locations in pigs. Our previous study showed that the IMF content of the longissimus thoracis muscle was greater than that of the semitendinosus muscle in 180-day-old pigs [6], and correlation analysis between IMF contents determined by the Soxhlet extraction method and computed tomography (CT) in vivo assessment in the longissimus thoracis, gluteal medius and semimembranosus muscles [7]. Although studies have shown that the type of pig muscle fiber can affect the IMF content, the molecular mechanism by which IMF formation is regulated by fast/slow muscles remains unclear.

Skeletal muscle is a highly heterogeneous secretory tissue that accounts for approximately 40%–60% of body weight and is composed of muscle fibers, muscle membranes, myosatellite cells, and other components, such as blood vessels, connective tissue, intramuscular adipocytes, and immune cells [8, 9]. Fleshy characteristics are affected by the composition of skeletal muscle fibers, especially the type, number and diameter of muscle fibers [10, 11]. According to the contraction characteristics, porcine skeletal muscle fibers can be classified as slow-twitch or fast-twitch muscle fibers. Normally, pork with a high content of slow-twitch muscle fiber shows better meat quality traits including meat color, pH, tenderness, flavor, juiciness and shear force [12, 13]. Therefore, elucidating the characteristics of skeletal muscle fibers and increasing the IMF content have become solutions for increasing pork quality. Skeletal muscle can secrete various muscle factors to regulate adipose tissue in a paracrine manner. Exosomes are vital intermediaries of material and information transfer between adipocytes and muscle cells in pork. Exosomes are tiny vesicles with a diameter of approximately 30–150 nm that can be excreted by cells and taken up by other cells [14]. Almost all cells can communicate with other cells or organs by releasing exosomes to transport part of their cellular contents to the extracellular space to be taken up by recipient cells, and skeletal muscle can also secrete exosomes [15]. The exosome content is complex and includes proteins, lipids, and RNA species [16]. Approximately 70% of the regulatory effects of exosomes on target cells are mediated by miRNAs, followed by proteins, which account for only 10% of the regulatory effects [17]. However, there is no information on the correlation between IMF traits and fast/slow muscle secreted exosomes. Due to the close anatomical location between myofibers and IMFs, exosomes may mediate cell-to-cell communication between these structures [18, 19]. To date, the effect of porcine fast/slow muscle exosomes on IMF deposition remains unclear. Therefore, this study aimed to 1) investigate skeletal muscle type and IMF content in the porcine gastrocnemius (GAS), tibialis anterior (TA), soleus (SOL) and extensor digitorum longus (EDL) muscles; 2) isolate and identify exosomes from the EDL and SOL muscles, which represent porcine fast/slow-twitch skeletal muscle; and 3) explore the effect of porcine fast/slow muscle exosomes on lipid accumulation in intramuscular adipocytes in a primary cell culture model.

Although many cytokines and metabolites have been reported to be involved in muscle-fat tissue crosstalk, further study of the systemic regulatory mechanism of exosome-mediated communication between muscle and adipose tissue is needed. In this study, we first isolated and identified 72 significantly differentially expressed proteins—31 proteins with a lower abundance and 41 proteins with a higher abundance in SOL-EXO. Interestingly, porcine SOL-EXO promoted lipid accumulation in longissimus intramuscular adipocytes by increasing the levels of key adipogenic proteins, including exosomal proteins such as FASN, but EDL-EXO had the opposite effect in vitro. The accumulation of lipids in intramuscular adipocytes can affect meat color, tenderness, and shear force and improve pork quality. Therefore, these findings provide new insight into the use of exosomes from fast/slow skeletal muscle to improve pork quality through skeletal muscle-fat interactions.

## Materials and methods

### Animals

The 8 Large White pigs, 4 3-day-old piglets and 4 180-day-old finishing pigs used in this study were all purchased from the experimental station of Northwest A&F University and handled in accordance with the guidelines of the Northwest A&F University Animal Care Committee. These animals were allowed access to feed and water ad libitum under the same feedstuff and management conditions and were ultimately humanely sacrificed to ameliorate suffering. SOL, GAS, TA and EDL samples were collected from 8 pigs at 3 and 180 days of age. The samples were immediately preserved in liquid nitrogen for preparation for histological slides and fluorescence double-standard staining of fast/slow skeletal muscle fibers, Oil Red O (ORO) staining and IMF content analysis.

### Porcine primary cell isolation and culture

SOL, EDL and LD skeletal muscles from 3-day-old Large White piglets were aseptically isolated, and all visible connective tissue and blood vessels were removed. After the tissues were minced to 0.5 mm^3^ in serum-free Dulbecco's modified Eagle’s medium (DMEM)/F12, 12,500 U of collagenase I/II was added to the finely minced tissues, and the samples were digested in a water bath at 37 °C for 1 h. After digestion, the isolated cells were neutralized with DMEM/F12 and passed through sterile 70 mm and 200 mm steel mesh filters. The cells were rinsed with serum-free DMEM/F12 medium, centrifuged twice at 1,500 × *g* for 10 min and resuspended in DMEM/F12. Viable cells were plated at a density of 6 × 10^5^ cells per 60-mm culture dish and cultured in a 5% CO_2_ incubator at 37 °C. Then, the cultured cells were rinsed with phosphate buffer saline (PBS) three times 1 h after seeding to remove insoluble myofibrillar proteins and other insoluble debris [6]. The cells were cultured in DMEM/F12 growth medium until they reached 80% confluence and then digested with 0.05% trypsin, which contained 0.5 mmol/L ethylenediaminetetraacetic acid (EDTA). The cells were collected by centrifugation at 1,500 × *g* for 5 min, resuspended in growth medium, plated at a density of 5 × 10^4^ cells/cm in a 6-well plate and used to induce differentiation.

At d 2 after the cells reached 100% confluence, the growth medium was replaced with induction medium consisting of DMEM/F12 supplemented with 10% fetal bovine serum (FBS), supplemented with 5 μg/mL (872 nmol/L) insulin, 1 μmol/L dexamethasone and 0.5 mmol/L isobutyl methylxanthine (IBMX, Sigma-Aldrich, St. Louis, MO, USA), for 2 d to induce differentiate. Then, the medium was removed, and the cells were cultured in DMEM containing 10% FBS and 1 μg/mL insulin for 2 d. The cells were then maintained in DMEM supplemented with 10% FBS, and the medium was changed every 2 d.

Porcine skeletal muscle satellite cells were isolated from the SOL and EDL muscles of 3-day-old piglets. Consistent with previous studies [20, 21], tissue block digestion was used to isolate skeletal muscle satellite cells in a sterile environment. Muscle tissue was cut into pieces (0.5 mm^3^) and then digested with 320 U/mL collagenase type II (Gibco, Los Angeles, CA, USA) in a 37 °C water bath with shaking for 1 h. After termination with F12 medium (Gibco, Los Angeles, CA, USA) supplemented with 10% FBS (Gibco, Los Angeles, CA, USA), the cell suspension was filtered through 70 mm and 200 mm steel mesh filters to remove tissue debris. The filtrate was retained. Afterward, the resulting cell pellet was resuspended and cultured in PM+ consisting of Roswell Park Memorial Institute (RPMI)-F12 medium supplemented with 20% FBS, 0.5% GlutaMax (Gibco, Los Angeles, CA, USA), 0.5% nonessential amino acids (Gibco, Los Angeles, CA, USA), 0.5% penicillin/streptomycin solution (Gibco, Los Angeles, CA, USA), 0.25% chicken embryo extract (Gemini, Woodland, CA, USA), and 2.5 ng/mL basic fibroblast growth factor (Invitrogen, Grand Island, NY, USA).

The isolated porcine SOL and EDL muscle satellite cells were induced to differentiate into myotubes. When the cells reached 70%–80% confluence, they were transferred to F12 medium supplemented with 5% horse serum (Gibco, Los Angeles, CA, USA) to induce differentiation, and the medium was changed every 2 d.

### 3T3-L1 and C2C12 cell culture

3T3-L1 cells (American Type Culture Collection^®^, CL-173) were cultured in DMEM supplemented with 1 g/L glucose (Gibco) and 10% FBS (Thermo Fisher Scientific), at 37 °C in a humidified CO_2_ incubator. For differentiation, cells were seeded at 30,000 cells/cm^2^ in the same media for differentiation and incubated for 48 h until they reached 100% confluence. Then, the cells were differentiated with 1× DMEM supplemented with 10% FBS, 0.25 μmol/L dexamethasone (Sigma‒Aldrich) and 0.5 mmol/L IBMX (Sigma‒Aldrich). The medium was changed to maintenance media (DMEM) 48 h after the initiation of differentiation, and the cells were refed with the same media every 2 d until d 8.

For culture of the C2C12 myoblast cell line, growth medium consisting of DMEM supplemented with 10% FBS, and 1% penicillin–streptomycin was used. The cells were then transferred to differentiation medium supplemented with 2% horse serum (Solarbio), 1% penicillin–streptomycin, and Dulbecco’s modified Eagle’s medium, which triggers differentiation, when the cell density reached 80% to 90%. The cells were incubated in a cell incubator at a fixed temperature and humidity (37 °C and 5% CO_2_), and the medium was replaced every 2 d.

### Fluorescence double-standard staining of muscle tissue

For staining of muscle sections, we collected porcine SOL, GAS, TA and EDL muscle samples frozen in liquid nitrogen-cooled isopentane in Tissue-Tek OCT and then sliced the muscles into 30 μm sections with a cryostat (CM1850, Leica) for staining [22]. The muscle sections were fixed with paraformaldehyde (PFA)/PBS (1%, 10 min), washed with PBS/0.1% Tween-20 for 3 × 5 min, and incubated with primary antibodies. The antibodies used included mouse anti-MyHC I (BA-D5-S 1:1,000, DSHB) and mouse anti-MyHC IIb (BF-F3 1:1,000, DSHB). The sections were washed in PBS/0.1% Tween-20 and incubated with Alexa Fluor-labeled (goat anti-mouse IgM/Alexa Fluor 555 antibody, bs-0368G-AF555, Bioss) and FITC-labeled (goat anti-mouse FITC, bs-50950, Biowarld) secondary antibodies (1:1,000, 1 h). The mounted slides were imaged on a fluorescence inverted microscope (Olympus). Each section was photographed using a microscope (*n *= 3), after which the number of muscle fibers of different colors was counted using ImageJ software. Fluorescence intensity images were captured with a fluorescence microscope (Nikon Eclipse C1, Japan) and analyzed using CaseViewer (3DHISTECH, Hungary). Slow MyHC-positive fibers were stained green, and fast MyHC-positive fibers were stained red. Then, ImageJ software was used to quantify the muscle fiber types. Three images were taken for each group. More than 150 muscle fibers were counted per image (scale bar represents 200 μm).

### PAX7/MyoD/MyHC immunofluorescence staining

After the cells were collected, the medium was discarded, and the cells were washed three times with PBS. The cells were fixed with 4% cold paraformaldehyde for 20 min and washed 3 times with PBS for 5 min/wash. The cells were permeated with 0.5% Triton X-100 for 10 min and washed with PBS 3 times for 5 min each time. The cells were blocked with 5% BSA for 30 min and washed with PBS 3 times for 5 min each time. The samples were incubated at 37 °C for 2 h and washed 3 times with PBS. PAX7 (1:1,000 dilution, CY5913, Abways), MyoD (1:1,000 dilution, CY8838, Abways), and MyHC (1:1,000 dilution, ab207926, Abcam) antibodies were incubated at room temperature for 2 h (in the dark) or at 37 °C for 1.5 h, after which the cells were washed with PBS 4 times for 5 min/wash. The sections were incubated with goat anti-rabbit IgG H&L (1:1,000 dilution, AB0121, Abways) at room temperature for 2 h (in the dark) or 37 °C for 1.5 h and then washed with PBS 4 times for 5 min/wash. After 4′,6-diamidino-2-phenylindole (DAPI) or Hoechst staining (10 min), the sections were washed with PBS 3 times for 5 min/wash, after which the fluorescent film was directly illuminated.

### Determination of the IMF and TG contents

The IMF contents of porcine SOL, GAS, TA and EDL samples were determined according to a previously published Soxhlet extraction method [23]. The triglyceride (TG) content in porcine intramuscular adipocytes was determined according to a previously published method [24, 25]. Briefly, the TG contents were determined on d 6 of adipocyte differentiation. The cells were digested with 0.25% trypsin and then homogenized by ultrasonic processing. The TG content in the lysates was measured using a TG test kit (Nanjing Jiancheng Bioengineering Institute, Nanjing, China) following the manufacturer’s protocol.

### Oil Red O staining

ORO staining was performed to identify lipid droplets resulting from porcine SOL, EDL, TA and GAS intramuscular adipocyte differentiation. The ORO stock solution was prepared by dissolving ORO powder (0.7 g) in 100% isopropanol (200 mL), stirring overnight, and filtering through a 0.22-μm syringe filter. Three parts of the ORO stock solution were mixed with two parts of deionized water to prepare an ORO working solution, which was then filtered through a 0.22-μm syringe filter. The cells were rinsed with PBS and fixed with 10% formalin for 1 h at room temperature. After fixation, the cells were washed with 60% isopropanol and dried thoroughly. Next, ORO working solution was added, followed by incubation for 10 min. The cells were washed 4 times with deionized water, and images were captured using a microscope. For quantitative analysis, ORO was eluted from the cells using 100% isopropanol, and the absorbance was measured at 500 nm.

### BODIPY and AdipoRed immunofluorescence staining

The amount of lipid accumulation in SOL, EDL and LD intramuscular adipocytes was assessed by BODIPY (Sigma, USA) and AdipoRed (Sigma, USA) staining, and the nuclei were assessed by DAPI staining as described previously [26]. The cells were observed and photographed with a TE2000-S fluorescence microscope (Nikon, Japan).

### Isolation and characterization of exosomes

Exosomes in the culture supernatant of porcine SOL and EDL myotubes were isolated by ultracentrifugation. The specific steps were as follows. After the culture supernatant was centrifuged at 200 × *g* for 10 min and 1,200 × *g* for 30 min, large debris and dead cells were removed. The supernatant was subjected to ultracentrifugation at 100,000 × *g* for 70 min. Finally, the cells were washed with PBS and ultracentrifuged for 70 min at 100,000 × *g*. We resuspended the pellets in 100 µL of PBS and stored them at −80 °C until further use.

### TEM and SEM analysis

The morphological characteristics of the exosomes were determined using transmission electron microscopy (TEM) and scanning electron microscopy (SEM). For TEM, in brief, exosomes were fixed with PBS (pH 7.4) containing 2% glutaraldehyde overnight, rinsed with PBS, and then fixed with 1% osmium tetroxide at 4 °C overnight. Then, the samples were dehydrated in a gradient of acetone and embedded in epoxy resin. Ultrathin sections were prepared and adsorbed onto formvar-coated copper grids. The samples were stained with uranyl acetate and lead citrate and then observed via TEM (Tecnai, USA). For SEM, a suspension of exosomes was mounted on aluminum holders covered with silver-pasted glass, freeze-dried, and then coated with platinum under a vacuum before analysis (S4800 U field emission scanning electron microscope; Hitachi, Tokyo, Japan).

### Nanoparticle tracking analysis (NTA) of exosomes

The exosomes were diluted to a ratio of 1:6 for subsequent research. The performance of the NanoFCM instrument (Flow NanoAnalyzer) was tested with standard products, and exosome samples were loaded after the standard was assessed. After the sample was tested, the particle size and concentration of the exosomes were determined.

### Raman spectroscopy analysis of exosomes

SOL-EXO and EDL-EXO isolated by size-exclusion chromatography were concentrated by ultracentrifugation (100,000 × *g* for 70 min). Then, the exosomes were analyzed by Raman spectroscopy (LabRAM, Horiba Jobin Yvon) following a previously published protocol [27].

### Proteomic analysis of exosomes

Protein extraction and digestion and liquid chromatography with mass spectrometry (LC–MS/MS) analysis were performed according to the literature [28]. For bioinformatics analysis, the MaxQuant software package (v1.6.6.0) was used to retrieve the secondary MS data, and the Swiss-Prot_Human data were used as a reference (Proteome ID: UP000005640). The sequences of the identified proteins were mapped according to Gene Ontology (GO) analysis to determine their biological function using InterProScan (v.5.14-53.0, http://www.ebi.ac.uk/interpro/). Protein enrichment pathway assessment was performed with the Kyoto Encyclopedia of Genes and Genomes (KEGG) database and the KAAS tool (v.2.0, http://www.genome.jp/kaas-bin/kaas_main). The identified proteins were compared with exosome-available data from the ExoCarta database (http://www.exocarta.org).

### PKH26 exosome uptake test

Freshly isolated SOL and EDL exosomes were labeled with the PKH67 Green Fluorescent Cell Linker Mini kit (MINI67, Sigma, USA) according to the manufacturer’s protocol. Briefly, exosomes diluted in PBS were added to 1 mL of Diluent C. In parallel, 4 μL of PKH67 dye was added to 1 mL of Diluent C and incubated with the exosome solution for 4 min. Labeling was stopped by the addition of 2 mL of 0.5% BSA/PBS. The labeled exosomes were centrifuged at 105,000 × *g* for 2 h, and the resulting pellet was diluted in 100 μL of PBS. PKH67-labeled exosomes were added to the culture medium of recipient cells growing on microscope coverslips and incubated at 37 °C for 30 min or 2, 4 or 24 h. Porcine LD intramuscular adipocytes were also incubated with PKH67-labeled exosomes for 12 h at 4 °C, and DAPI was used as a nuclear marker.

## Western blot analysis

Total protein was isolated from cells by incubation in RIPA buffer containing a protease inhibitor cocktail for 30 min and then centrifugation at 12,000 × *g* for 10 min at 4 °C to remove the precipitate. The total protein concentration was determined using the BCA Protein Assay kit (Pierce), and 30 μg of protein was separated by reducing SDS‒PAGE on 10% or 15% Bis‒Tris gels, transferred to PVDF membranes (EMD Millipore) and probed with different primary antibodies:anti-TSG101 (1:1,000 dilution, ab30871, Abcam), anti-CD9 (1:500 dilution, sc-13118, Santa Cruz, CA, USA), anti-CD63 (1:500, sc-5275, Santa Cruz, CA, USA), anti-CD81 (1:500, sc-166029, Santa Cruz, CA, USA), anti-PPARγ (1:1,000, ab17860, Abcam), anti-CEBPβ (1:1,000, ab32358, Abcam), anti-FABP4 (1:1,000, ab92501, Abcam), anti-FASN (1:1,000, ab128870, Abcam), anti-ATGL (1:1,000, sc-365278, Santa Cruz, CA, USA), anti-HSL (1:1,000, ab45422, Abcam), anti-MINK1 (1:500, NBP1-22989, Novus), anti-PIN1 (1:500, SC-46660, Santa Cruz, CA, USA), anti-SUMO1 (1:1,000, ab32058, Abcam) and anti-β-tubulin (1:1,000, sc-166729, Santa Cruz, CA, USA) at 4 °C overnight. Then, the cells were incubated with a goat anti-mouse IgG secondary antibody (1:1,000, 14709, CST) or a goat anti-rabbit IgG secondary antibody (1:1,000, 14708, CST) for 1 h at room temperature. The protein bands were visualized using a Gel Doc XR System (Bio-Rad, Richmond, CA, USA). and analyzed using ImageJ software. The data were normalized to the expression of β-tubulin.

### H/E staining and determination of muscle fiber size

Briefly, sections were deparaffinized in xylene (2 × 5 min) and rehydrated with successive 1-min washes in 100%, 96%, 80%, and 70% ethanol. The sections were then stained with hematoxylin (2 min), rinsed with distilled water, rinsed with 0.1% hydrochloric acid in 50% ethanol, rinsed with tap water for 15 min, stained with eosin for 1 min, and rinsed again with distilled water. The slides were then successively dehydrated with 95% and 100% ethanol, followed by xylene (2 × 5 min) and mounting with coverslips. The muscle fiber size/cross-sectional area was quantified using ImageJ software.

### L*, a* and b* measurements

A color difference meter (3nh, China) was used to determine the values of L*, a* and b*. Briefly, the lens of the color difference meter was vertically placed on the cross section of the muscle for measurement, and the mirror mouth was close to the muscle (no light leakage). Each sample was measured 5 to 8 times, the values of L*, a* and b* were recorded.

### Statistical analysis

Diagrams were generated using GraphPad Prism version 8.0 statistical software (La Jolla, CA, USA), and all the data are presented as the mean ± SEM. The significance of differences between the groups was assessed using one-way analysis of variance (ANOVA) and multiple *t*-tests. *P* values less than 0.05 were considered to indicate statistical significance, and different lowercase letters indicate significant differences.

## Results

### Myofiber composition and IMF contents in porcine SOL, GAS, TA and EDL samples

**Fig. 1:**
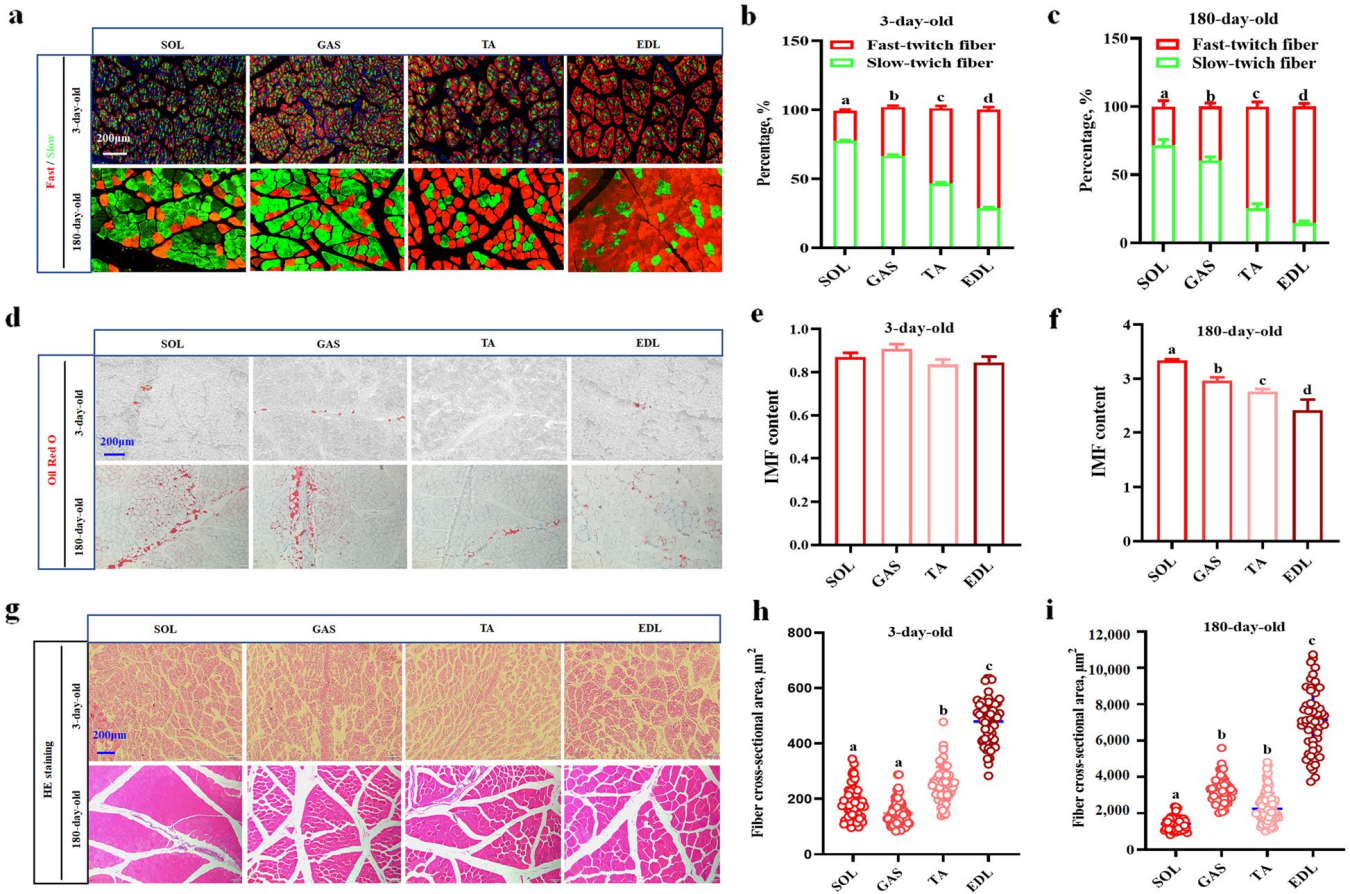
Differences of fast/slow muscle fiber and IMF content in porcine SOL, GAS, TA and EDL. **a** Fluorescence double-standard staining of fast/slow skeletal muscle fiber in porcine SOL, GAS, TA and EDL on 3 and 180 days of age, Red: fast MyHc, Green: slow MyHc. **b** and** c** Analysis of fast/slow muscle fiber number. For counting fast/slow muscle fiber number, samples from 3 randomly selected pigs in each group and data from 3 pigs in each group were compared. **d** ORO staining. **e** and** f** IMF content analysis, 3 randomly selected from pigs in each group were compared. **g** HE staining. **h** and** i** Analysis of fiber cross-section area. For counting the cross-section area of muscle fiber, 3 randomly selected images in each sample and data from 3 pigs in each group were compared. ^a^^–^^d^Different lowercase letters indicate significant differences (*P* < 0.05)

We investigated the correlation between myofiber type and the IMF content of porcine skeletal muscle. To study the differences in the composition and IMF content of muscle fiber types in different muscle tissues of pigs, we collected SOL, GAS, TA and EDL muscle tissue samples from 3-day-old and 180-day-old pigs for analysis. As shown in Fig. 1a, the number of slow-twitch muscle fibers was greatest in the SOL group, but the number of fast contractile muscle fibers in the EDL samples predominated at 3 days of age and likewise tended to increase at 180 days of age (Fig. 1b). Compared with those at 3 days of age, the numbers of fast-twitch muscle fibers in the GAS, TA and EDL samples apparently increased at 180 days of age (Fig. 1c). Interestingly, using ORO staining (Fig. 1b), we found that the SOL, GAS, TA and EDL samples contained trace amounts of IMF when the animals were 3 days of age (Fig. 1d and e). However, in the 180-day-old pigs, the IMF content in the four skeletal muscles increased by 3–8 times and was greatest in the SOL muscle (Fig. 1d–f). Moreover, the muscle fiber cross-sectional area in the EDL muscle was greatest among the four muscles in both the 3 and 180 d samples, whereas it was the lowest in the SOL muscle (Fig. 1g–i). Compared with that in the 3 d samples, the muscle fiber cross-area in the EDL samples increased by 14 times at 180 days of age (Fig. 1g–i).

### Porcine SOL adipocytes have a greater lipid accumulation capacity than do EDL adipocytes

**Fig. 2:**
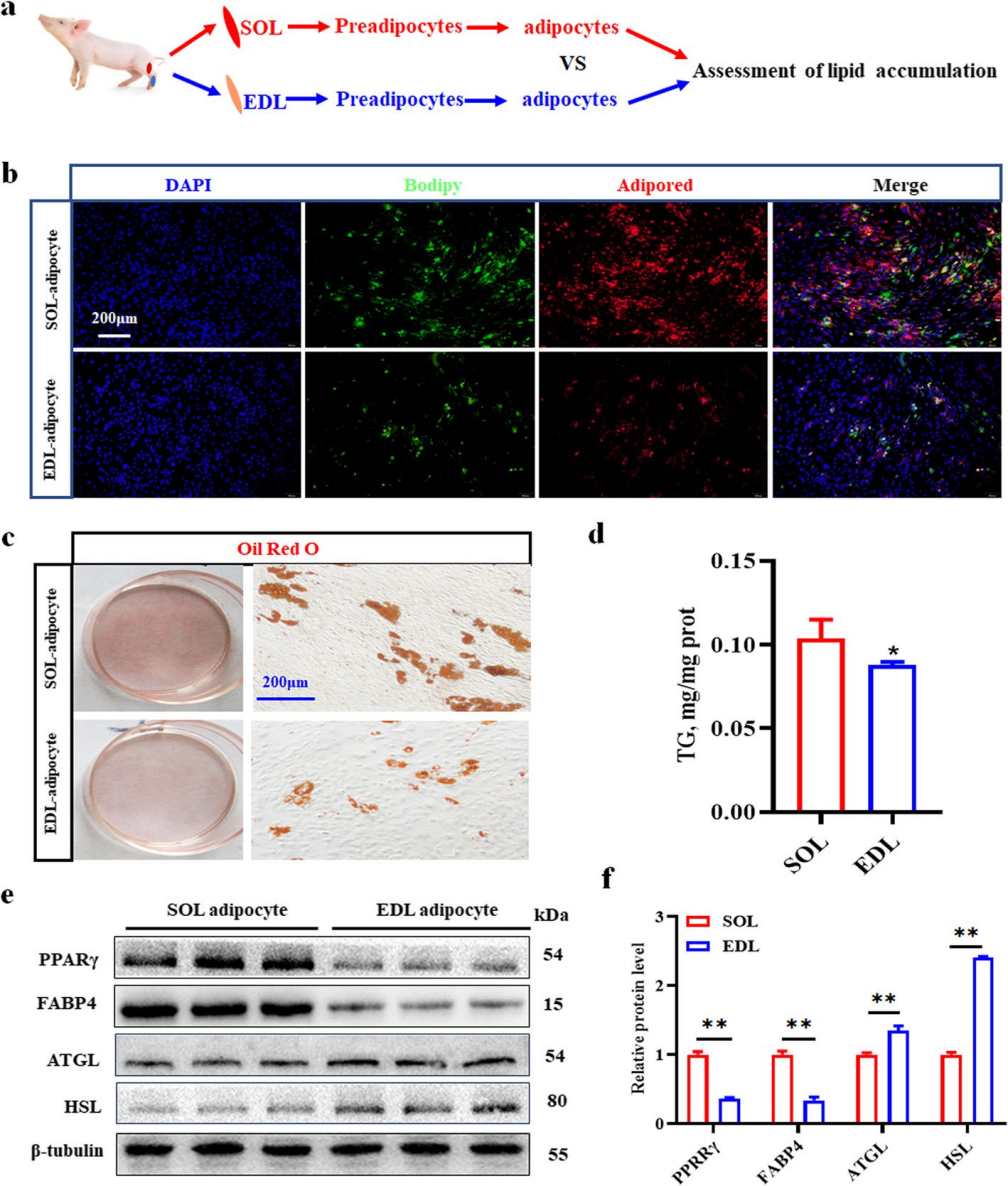
SOL intramuscular adipocytes have a stronger lipid accumulation than that of EDL in vitro. **a** Flow diagram on identification of porcine SOL and EDL intramuscular adipocytes. **b** Bodipy and adipored staining of SOL and EDL intramuscular preadipocytes after induction on d 6, Red: adipored staining, Green: Bodipy staining. **c** ORO staining. **d** TG contain analysis. 3 randomly selected from pigs in each group were compared. **e** Western blotting detection of the key lipogenic and lipolytic proteins. **f** The relative protein levels of PPARγ, FABP4, ATGL and HSL. ^*^P < 0.05; ^**^P < 0.01

For determination of significant differences in the IMF contents between porcine SOL and EDL samples, intramuscular preadipocytes were isolated, cultured and induced to undergo adipogenic differentiation (Fig. 2a). The SOL and EDL of 3-day-old piglets were separated (Fig. S1a), after which proliferation and differentiation were successfully induced in the SOL and EDL preadipocytes (Fig. S1b). The results showed that the fluorescence intensity (green or red light) in the SOL adipocytes was significantly greater than that in the EDL adipocytes according to BODIPY and AdipoRed immunofluorescence staining on d 6 after induction (Fig. 2b). Similarly, by ORO staining, we found that SOL adipocytes had significantly greater lipid droplet and TG contents than did EDL adipocytes (Fig. 2c and d). Moreover, western blot analysis revealed that the protein levels of the key adipogenic genes PPARγ and FABP4 were greater (*P* < 0.05) in SOL adipocytes than in EDL adipocytes, whereas the protein levels of the lipolytic genes HSL and ATGL were lower (*P* < 0.05) in SOL adipocytes than in EDL adipocytes (Fig. 2e and f). Therefore, porcine SOL adipocytes have a greater capacity for lipid accumulation than do EDL derived adipocytes, resulting in a significant difference in the IMF content between porcine SOL and EDL.

### Isolation and identification of porcine SOL and EDL-derived exosomes

**Fig. 3:**
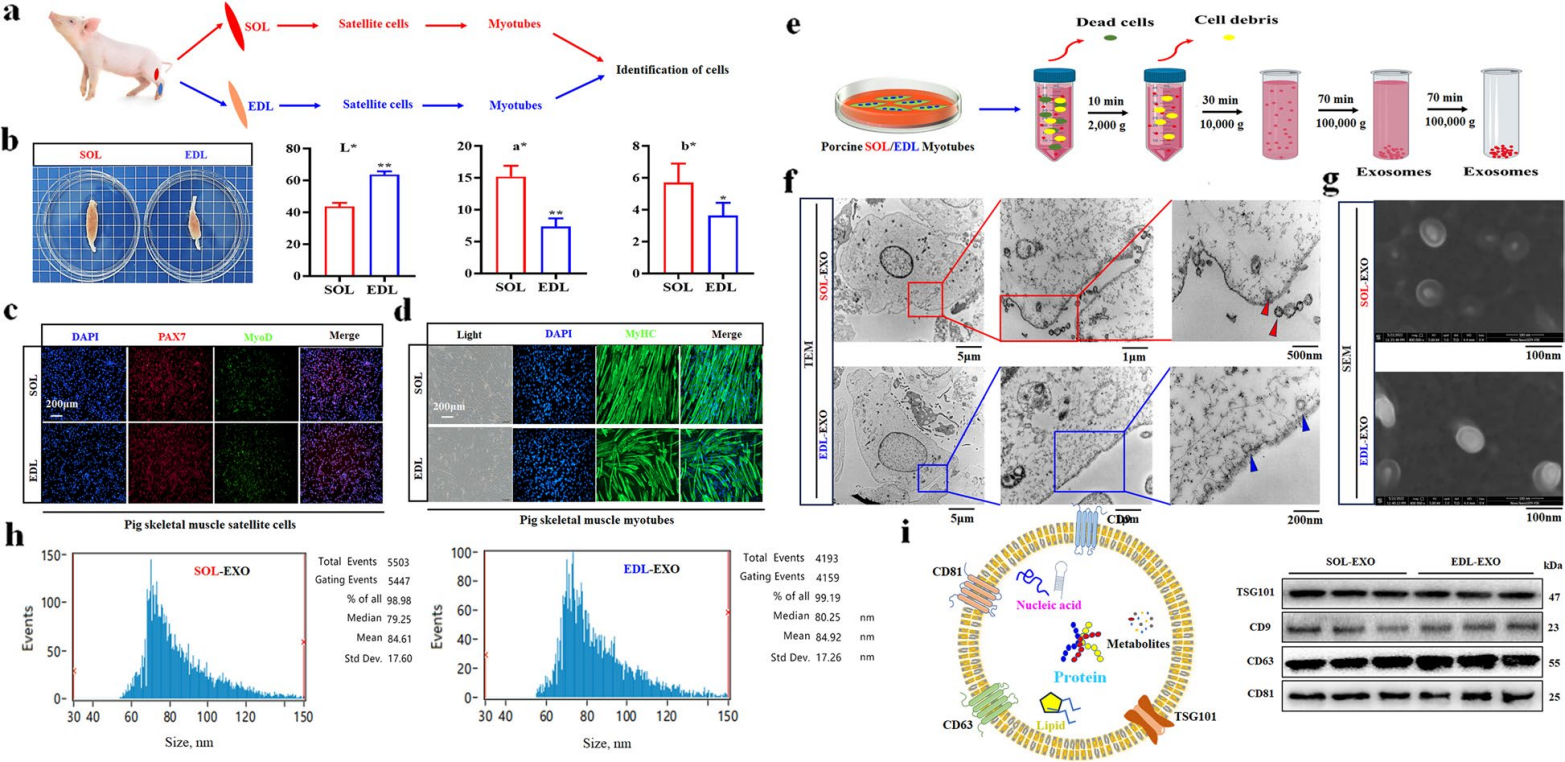
Isolation and identification of porcine SOL and EDL myotube-derived exosomes. **a** A flow chart on cultured SOL and EDL myotubes. **b** The SOL and EDL of piglet on 3 days of age. **c** Identification of SOL and EDL satellite cells, Red: PAX7, Green: MyoD.** d** Identification of SOL and EDL myotubes. **e** A flow chart on obtained SOL and EDL myotube-derived exosomes (SOL-EXO and EDL-EXO). **f** and **g** Observation of exosomes by TEM and SEM. **h** NTA Analysis of exosome size. **i** Western blot detection of exosome membrane proteins. ^*^P < 0.05; ^**^P < 0.01

First, porcine SOL and EDL satellite cells and myotubes were isolated and identified (Fig. 3a). The SOL and EDL muscles of 3-day-old pigs were sampled, and we found that the L* value of the SOL sample was greater than that of the EDL sample, but the a* value and b* value of the SOL sample were significantly lower (Fig. 3b). Moreover, the cells isolated from the SOL or EDL samples were identified as skeletal muscle satellite cells using paired box 7 (PAX7) and myogenic differentiation 1 (MyoD) immunofluorescence staining during proliferation at d 1 (Fig. 3c). Similarly, the induced cells were determined to be pig skeletal muscle myotubes by MyHC immunofluorescence staining during differentiation at d 2 (Fig. 3d).

Second, porcine SOL- and EDL-derived exosomes were isolated and identified (Fig. 3e). TEM clearly showed that both the SOL myotubes and EDL myotubes secreted exosomes from the plasma membrane, some of which had completely left the membrane surface, as indicated by red or blue triangles, respectively (Fig. 3f). Moreover, under a scanning electron microscope, the SOL-EXO and EDL-EXO had a disc shape (Fig. 3g). The average sizes of the SOL-EXO and EDL-EXO were 84.61 and 84.92 nm, respectively, as determined by NTA of the exosomes (Fig. 3h), indicating that there was almost no difference in average particle size or shape between the SOL-EXO and EDL-EXO.

Finally, marker membrane proteins of porcine SOL-EXO and EDL-EXO were assessed by western blotting. The results showed that the marker membrane proteins in exosomes, including TSG101, CD9, CD63 and CD81, were expressed in the porcine SOL-EXO and EDL-EXO (Fig. 3i). In summary, porcine SOL-EXO and EDL-EXO were successfully isolated for the first time and identified for subsequent functional studies.

### Component analysis of SOL-EXO and EDL-EXO

**Fig. 4:**
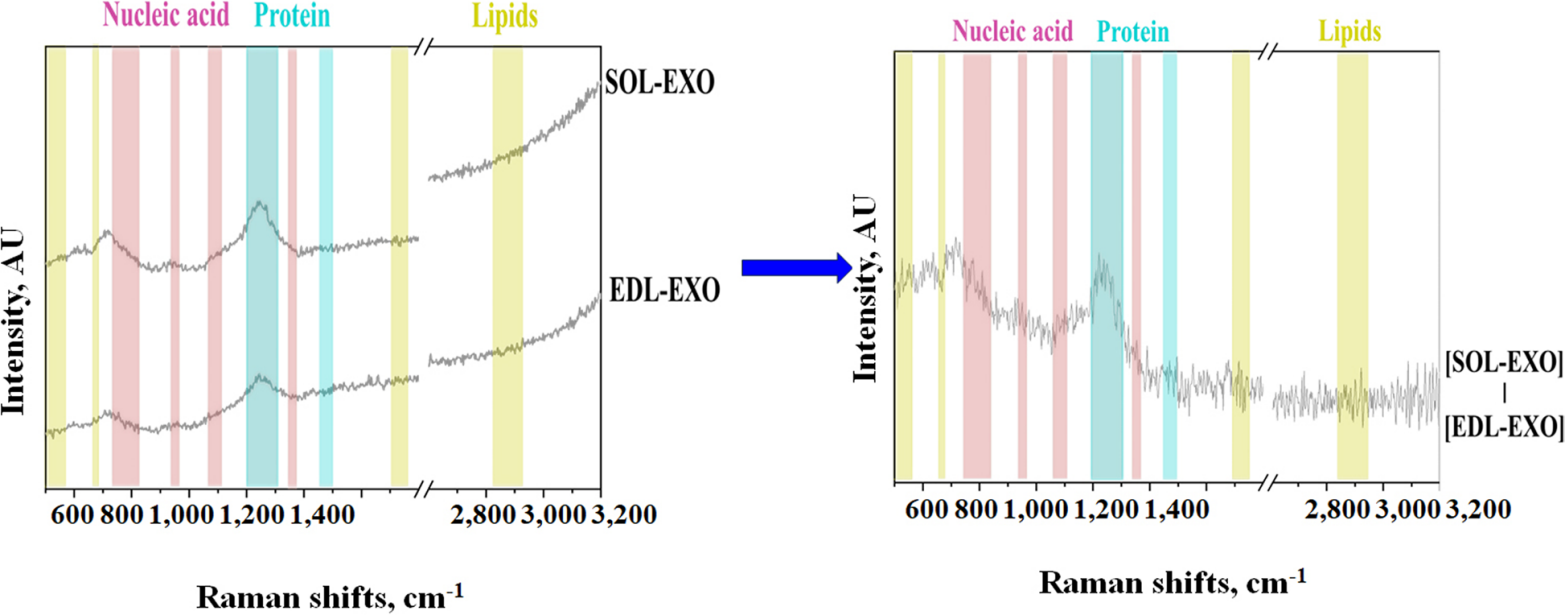
The Raman spectrum analysis between porcine SOL-EXO and EDL-EXO. The figure on the left shows the nucleic acid, protein and lipid content of SOL-EXO and EDL-EXO respectively, and the figure on the right shows the Raman spectral difference of SOL-EXO and EDL-EXO

To determine the components of the SOL-EXO and EDL-EXO, we performed Raman spectroscopy analysis. A schematic diagram of exosome components is shown in Fig. 3i. Raman spectroscopy in the spectral ranges of 500–1800 and 2600–3200 are the most important regions in biological specimens for Raman spectroscopy. In this study, Raman spectroscopy was used to analyze the nucleic acid, protein and lipid contents of the SOL-EXO and EDL-EXO. The nucleic acid-corresponding Raman bands are highlighted in red, the protein bands are highlighted in blue, and the lipid bands are highlighted in yellow. The results showed that both the SOL-EXO and EDL-EXO contained nucleic acids, proteins and lipids, and the protein content showed the greatest difference between the SOL-EXO and EDL-EXO (Fig. 4).

### Proteomic analysis of SOL-EXO and EDL-EXO

**Fig. 5:**
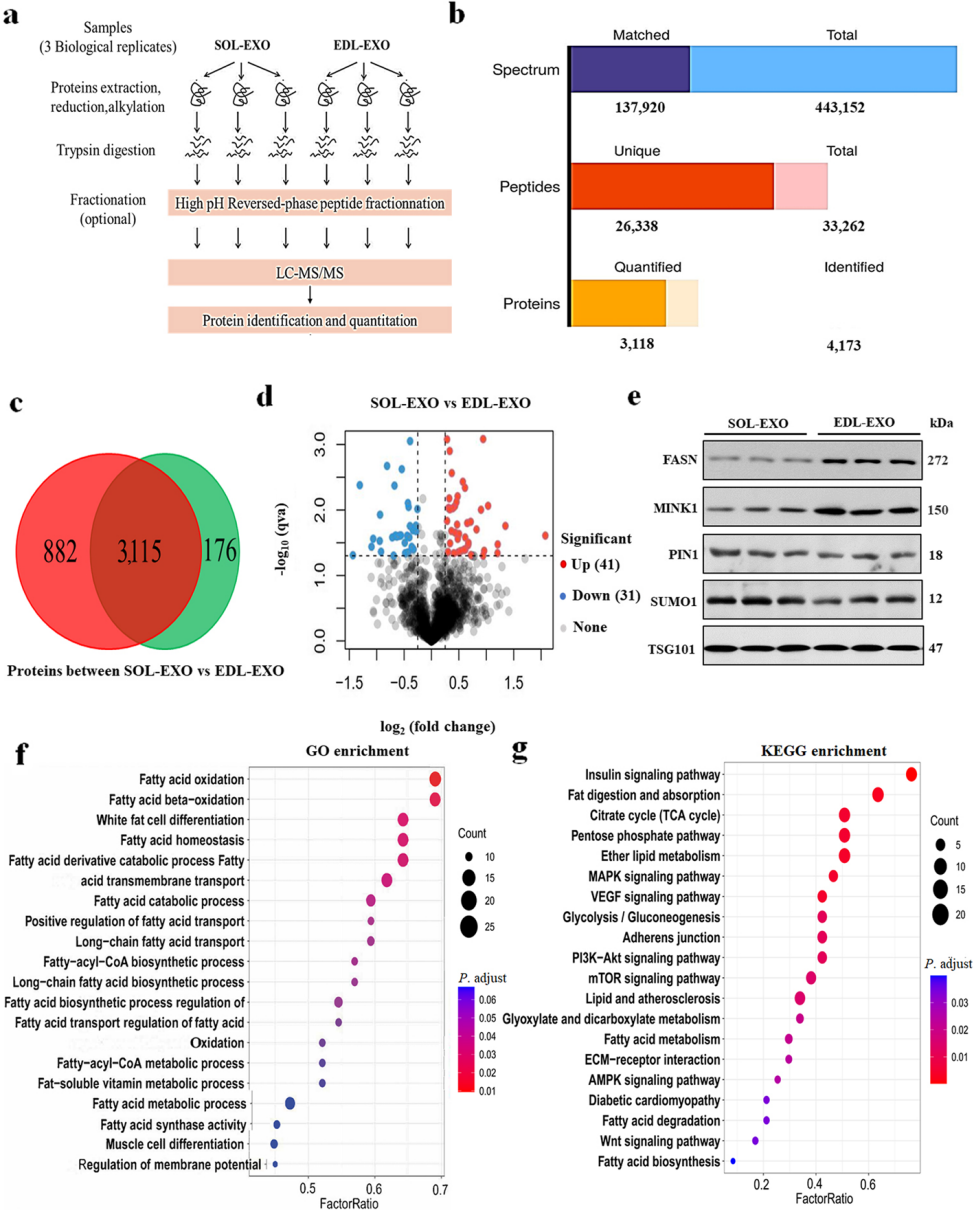
The differentially expressed protein analysis between porcine SOL-EXO and EDL-EXO. **a** A procedure of LC/MS on SOL-EXO and EDL-EXO. **b** The number of spectrum, peptides and proteins. **c** The number of differentially expressed proteins. **d** The differentially expressed protein analysis using volcano plot. **e** Western blot detection of FASN, MINKI, PIN1,TSG101 and SUMO1. **f** GO enrichment analysis. **e** KEGG enrichment analysis

To further explore the differences in protein content between the SOL-EXO and EDL-EXO, we performed 4D-label-free quantitative proteomics analysis (Fig. 5a). Bioinformatics analysis of the retrieved data revealed that a total of 33,262 peptides and 4,173 proteins were identified in this study, and a total of 3,118 proteins were quantified from the SOL-EXO and EDL-EXO (Fig. 5b). The relative molecular masses of these proteins ranged from 10 to 70 kDa (Fig. S2a). The Wayne plot showed that 3115 proteins were detected, but 882 proteins were specifically detected in the SOL-EXO, and 176 proteins were specifically detected in the EDL-EXO (Fig. 5c). The volcano plot indicated that a total of 72 proteins were differentially expressed between porcine SOL-EXO and EDL-EXO, of which 41 were more abundant in the SOL-EXO and 31 were less abundant in the SOL-EXO (Fig. 5d). Sixteen percent of the differentially expressed proteins were originally located in the cytomembrane, 12% in the nucleus and 10% in the cytoplasm (Fig. S2b). Compared with EDL-EXO, the top 10 up-regulated and down-regulated proteins of SOL-EXO are shown in Table S1 and Table S2, respectively. Furthermore, western blot analysis revealed that the levels of the differentially expressed proteins FASN and MINK1 were increased, but the levels of SUMO1 and PIN1 were decreased (Fig. 5e).

To fully elucidate the biological function of the proteins differentially expressed between porcine SOL-EXO and EDL-EXO, we annotated 72 proteins screened by GO analysis (Fig. 5f). GO and KEGG enrichment analyses revealed that the proteins identified in the SOL-EXO and EDL-EXO were involved in the secretion and formation of exosomes, among which the proteins highly expressed in the SOL-EXO, especially FASN, MINK1, PIN1 and SUMO1, were involved in the differentiation and lipid deposition of preadipocytes.

To more systematically analyze the biological signaling pathways associated with the 72 proteins, we identified the pathways enriched in the proteins by KEGG analysis (Fig. 5g). GO and KEGG enrichment analyses indicated that the porcine proteins of the SOL-EXO and EDL-EXO participated in the secretion and formation of exosomes, and the proteins detected in the SOL-EXO, especially FASN, MINK1, PIN1 and SUMO1, were implicated in preadipocyte differentiation and lipid deposition.

### SOL-EXO promote lipid accumulation in intramuscular adipocytes, but EDL-EXO inhibit lipid accumulation

**Fig. 6:**
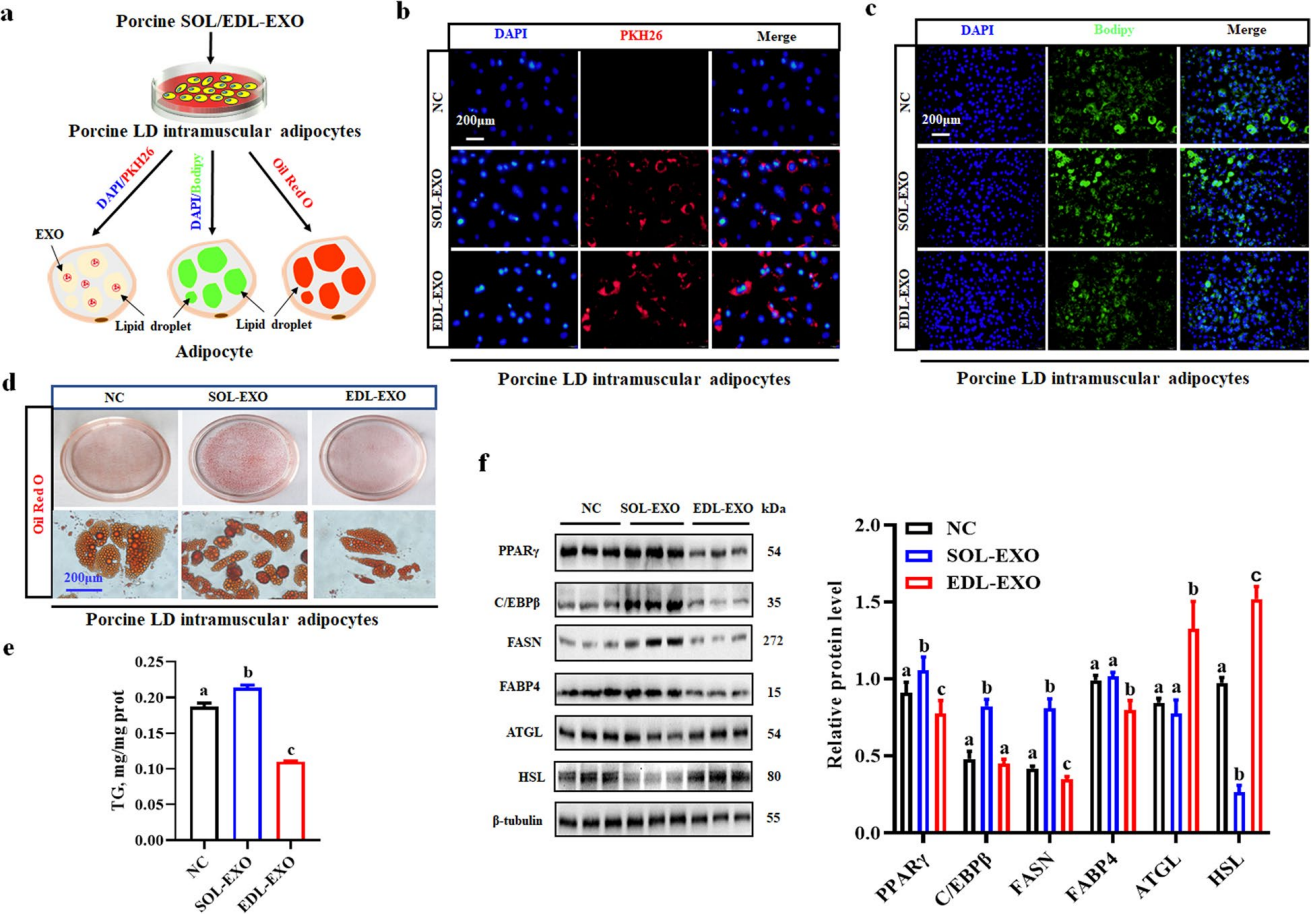
Porcine SOL-EXO improves lipid accumulation of LD intramuscular adipocytes, whereas EDL-EXO inhibits in vitro. **a** A mode of SOL/EDL-EXO modulating lipid accumulation of LD intramuscular adipocytes. **b** LD intramuscular adipocytes uptake exosomes by PKH26 immunofluorescence method, Blue: DAPI, Red: PKH26-label Exosome. **c** Bodipy saining. **d **and **e** Oil Red O (ORO) staining and triglyceride (TG ) content analysis, samples from 3 randomly selected pigs in each group. **f** Detection and analysis of western blot. ^a–c^Different lowercase letters indicate significant differences (*P* < 0.05)

**Fig. 7:**
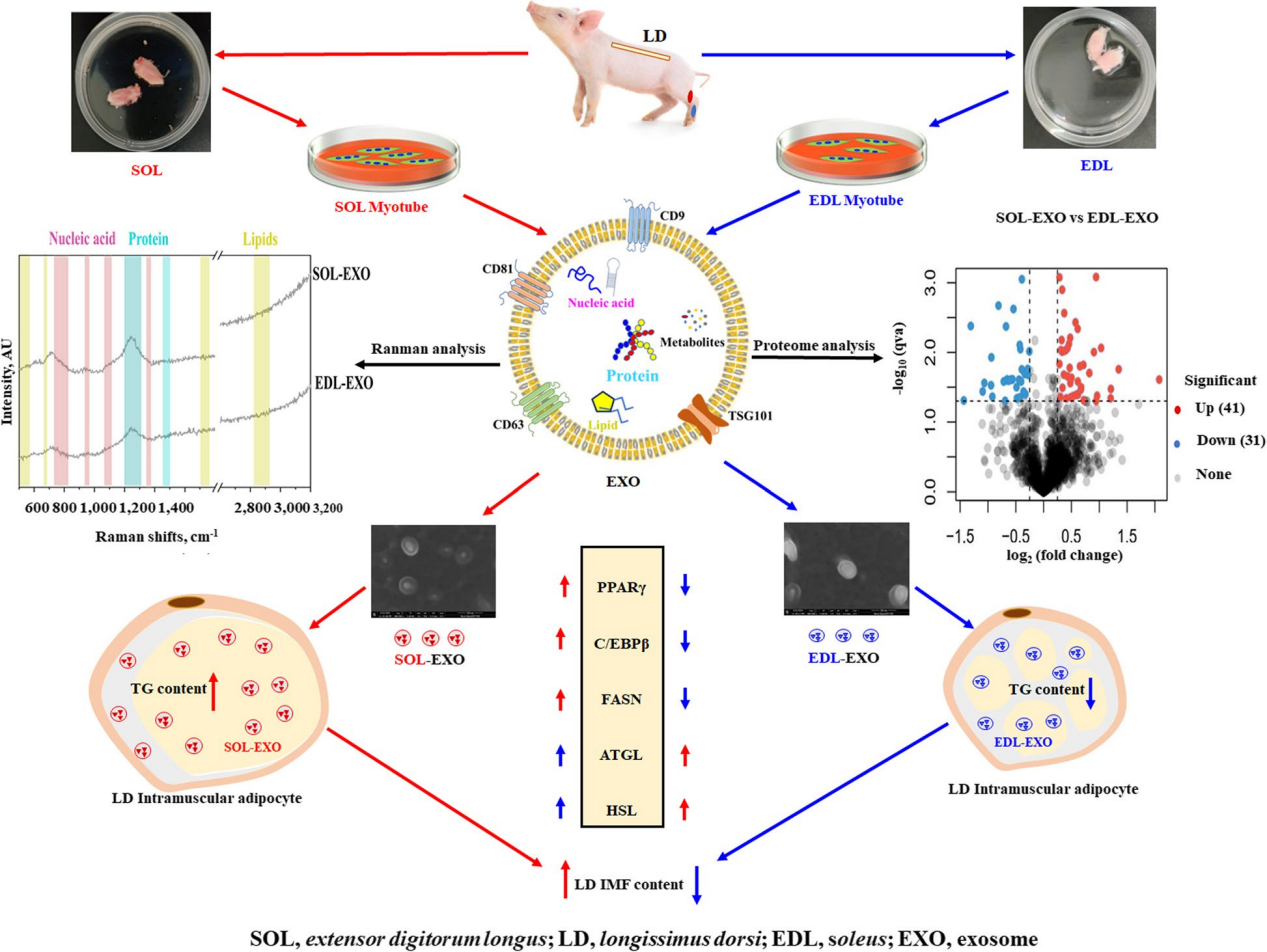
A diagrammatic illustration represents identification of porcine SOL/EDL-EXO and their adipogenic function on LD intramuscular adipocytes in vitro

Here, we obtained exosomes from C2C12 myoblasts and myotubes (Fig. S3a) and found that they both inhibited adipogenesis in 3T3-L1 preadipocytes, but the inhibitory effect of myotube-derived exosomes was greater (Fig. S3b and c). For analysis of the effects of the SOL-EXO and EDL-EXO on IMF deposition, porcine LD intramuscular adipocytes treated with NC (PBS), SOL-EXO or EDL-EXO were stained with PKH26, BODIPY or ORO (Fig. 6a). In this study, exosomes isolated from 50 mL of the supernatants of myotube culture with SOL- or EDL-induced differentiation of myotube culture supernatants were diluted with PBS to 100 μL for subsequent experiments. According to the PKH26 exosome uptake test assay, the SOL-EXO and EDL-EXO, which are marked with PKH26 red fluorescence, entered LD intramuscular adipocytes and were uniformly distributed in the cytoplasm after treatment for 24 h (Fig. 6b). BODIPY immunofluorescence staining showed that lipid droplets in the intramuscular adipocytes treated with the SOL-EXO had a stronger fluorescence intensity than those in the adipocytes treated with the EDL-EXO (Fig. 6c). Moreover, at 6 d after induction of differentiation, lipid droplets were stained with ORO, and the absorbance at 510 nm was determined via isopropanol extraction. The results showed that the LD intramuscular adipocytes treated with the SOL-EXO had a greater (*P* < 0.05) TG content but a lower (*P* < 0.05) TG content after the EDL-EXO treatment (Fig. 6d and e). Moreover, compared with those in the control group, the protein levels of the vital lipogenic genes *PPARγ*, *CEBPβ* and *FASN* were significantly increased in the adipocytes treated with the SOL-EXO, but the protein levels of the key lipolytic genes *ATGL* and *HSL* were markedly decreased (Fig. 6f). Conversely, compared with those in the control cells, the protein levels of PPARγ and FASN were significantly decreased in the cells treated with the EDL-EXO, whereas the protein levels of the lipolytic genes *ATGL* and *HSL* were sharply increased (Fig. 6f). Therefore, the SOL-EXO promoted lipid accumulation in intramuscular adipocytes, but the EDL-EXO inhibited adipogenesis. Figure 7 shows a diagrammatic illustration of the identification of porcine SOL/EDL-EXO and their opposite adipogenic effects on LD intramuscular adipocytes.

## Discussion

Improving meat quality through exosome-mediated muscle-fat tissue crosstalk is important [19]. In this study, we explored the reason why the IMF content of SOL was greater than that of EDL. Furthermore, porcine SOL-EXO and EDL-EXO were first isolated and identified, and differences in their proteins and nucleic acids were detected via Raman analysis. Seventy-two differentially expressed proteins were further identified via proteomic analysis. Importantly, the SOL-EXO promoted lipid accumulation in LD intramuscular adipocytes, but the EDL-EXO inhibited lipid accumulation in vitro. The accumulation of lipids in intramuscular adipocytes is believed to improve the tenderness, color and water content of pork, thus increasing pork quality. These findings provide new insight into the use of exosomes from fast/slow skeletal muscle to increase the IMF level via skeletal muscle-fat tissue crosstalk and indicate that these exosomes can be used as a means to improve pork quality.

Fast and slow muscles have different metabolic characteristics due to different muscle fiber types, which may affect the adipogenic capacity of adjacent intramuscular adipocytes [29, 30]. Fibro-adipogenic progenitors (FAPs), the cellular source of IMF and fibrotic scar tissue, are located in the interstitial space between muscle fibers and eventually result in the deposition of IMF [31–34]. Our previous study revealed that lipid accumulation in intramuscular preadipocytes derived from pig longissimus thoracis occurred earlier and was greater than that in SOL intramuscular preadipocytes, resulting in a much greater IMF content in 180-day-old pig LD muscle than in pig SOL muscle [6]. In the present study, we first confirmed that SOL intramuscular adipocytes had greater fat accumulation than did EDL intramuscular adipocytes because of the higher protein levels of key lipogenic genes and the lower protein expression of vital lipolytic genes, which may result in SOL having a greater IMF content than EDL. Why do SOL intramuscular adipocytes have a stronger lipogenic ability than EDL intramuscular adipocytes? Based on the above findings, we further speculated that the presence of fast and slow myogenic exosomes was important.

Exosomes are nanosized vesicles that are released by cells through phagocytosis and exocytosis and are involved in intercellular communication through loaded proteins and nucleic acids [35]. Through this process, exosomes mediate cell‒cell and organ‒organ communication, suggesting that skeletal muscle cells can also secrete exosomes that function in targeting cells [36]. Both muscle cells and fat cells belong to the mesoderm cell lineage, and the fact that these cells have the same origin indicates that there may be special interactions between muscle and fat tissue [37]. Moreover, this phenomenon may be due to the anatomical position of muscle fibers and IMF cells that are relatively close to muscles and because secretory organs show intramuscular crosstalk with adipocytes in the form of exosomes. Previous studies have shown that skeletal muscle can regulate the physiological and metabolic processes of adipose tissue by secreting exosomes. LD muscle-derived exosomes can inhibit the adipogenesis of porcine subcutaneous preadipocytes [38]. Furthermore, skeletal muscle-derived exosomal miR-146a-5p was shown to inhibit adipogenesis by mediating the muscle-fat axis and targeting growth differentiation factor 5 (GDF5)-PPARγ signaling [39]. However, the effect of different muscle fiber types and their secreted exosomes on the adipogenesis of intramuscular adipocytes remains unclear. In addition, LD, as a typical mixed muscle, is relatively less affected by different types of muscle fibers, and can be used as a typical model of intramuscular adipogenesis differentiation. In this study, porcine SOL-EXO and EDL-EXO, which were first identified, were added to LD intramuscular adipocytes to explore the effect of exosomes secreted by porcine fast and slow muscles on IMF accumulation. This study provides a new idea for exploring exosome-mediated muscle-fat tissue crosstalk to improve pork quality.

Most exosome biogenesis patterns tend to show a “delayed process” in which vesicles first germinate into an intermediate structure, which is subsequently released upon endosomal-plasma membrane fusion [40]. Here, we first found that both porcine SOL and EDL myotubes preferentially secrete exosomes as the plasma membrane buds outward. For exosomes in the C2C12 myotube culture supernatant, TEM analysis showed the generation and release of nanovesicles via direct budding from the plasma membrane [41]. This method of germinating directly from the plasma membrane is known as the “immediate mode” of exosome biogenesis, in contrast to the classical view of exosome release [42]. Furthermore, Raman spectroscopy in the spectral ranges of 500–1,800 and 2,600–3,200 is the most important for biological specimens [27]. In this study, according to the corresponding peaks of nucleic acids, proteins and lipids, Raman spectroscopy was used to analyze their contents in the freshly isolated SOL-EXO and EDL-EXO. We found that the SOL-EXO contained more nucleic acids, proteins and lipids than the EDL-EXO and speculated that the apparent difference in the protein content of exosomes may play an important role in targeting cells. In the present study, proteomic analysis revealed the top 10 significantly differentially abundant proteins between porcine SOL-EXO and EDL-EXO, among which fatty acid synthase (FASN), G protein-coupled receptor class C group 5 member B (GPRC5B), misshapen-like kinase 1 (MINK1)/cellular communication network factor 2 (CCN2), MacroH2A1, PIN1 and small ubiquitin-like modifier 1 (SUMO1) were implicated in adipogenesis. In the de novo synthesis pathway of fatty acids, the key rate-limiting enzyme FASN catalyzes the polymerization of small-molecule dicarbon units into long-chain fatty acids by acetyl CoA and malonic acid monoacyl CoA, contributing to adipocyte TG storage [43]. *GPRC5B* knockout in mice reduced fat production and prevented diet-induced obesity and insulin resistance [44]. The final step of cell division is physical disconnection between the two daughter cells via shrinking of the cleavage groove and detachment of the membrane, while MINK1 is a potential mitotic kinase necessary for final membrane shedding [45]. *CCN2* regulates CCAAT/enhancer binding proteins and inhibits fat cell differentiation through TGF-β signaling [46]. Overexpression of *MacroH2A1.2* inhibited adipogenesis, while knockdown of *MacroH2A1.1* had the opposite effect [47]. *PIN1* increases adipocyte differentiation by positively regulating the transcriptional activity of *PPARγ* [48]. SUMO1 increases adipogenesis through the EDL of Sharp-1 [49]. Based on the above analysis, the porcine SOL-EXO and EDL-EXO proteins are involved in lipid metabolism by participating in preadipocyte proliferation, differentiation, autophagy, and ubiquitination of related transcription factors. Therefore, the differentially expressed proteins of porcine fast and slow myogenic exosomes may provide a different functional advantage to these recipient cells, such as intramuscular adipocytes. However, the role of nucleic acids and lipids in exosomes cannot be ignored. Therefore, transcriptomic and lipidomic analyses can be further used for joint analysis to explore the differential expression of nucleic acids and lipids between SOL-EXO and EDL-EXO in the future.

Autocrine and paracrine intercellular communication is vital for regulating the functionality of a tissue or organ. Exosomes are specialized cargo delivery vesicles secreted from cells by the fusion of multivesicular bodies with the plasma membrane [40]. In this study, PKH26 staining, BODIPY immunofluorescence staining and ORO staining revealed that SOL-EXO entered LD intramuscular adipocytes and promoted adipogenesis, while EDL-EXO inhibited lipid accumulation. PPARγ is indispensable for adipogenic programming and is the master regulator of adipogenesis [50], and C/EBPβ can trigger the transcription of C/EBPα and PPARγ, which in turn coordinately induce the transcription of adipogenic genes, including *FASN* and *FABP4,* to promote adipogenic differentiation [51, 52]. Moreover, the major enzymes participating in lipolysis are ATGL and HSL [53, 54]. Here, SOL-EXO increased the levels of key adipogenic proteins, including C/EBPα, PPARγ, FASN and FABP4, in LD intramuscular adipocytes, resulting in accelerated TG synthesis and downregulating the protein expression of the above vital lipolytic genes and the key lipolytic genes encoding *ATGL* and *HSL*, thereby inhibiting TG lipolysis. Interestingly, the opposite results were found for EDL-EXO. The literature has shown that single myofiber SOLs contain more intramyocellular lipids than do single myofiber EDLs due to greater mRNA expression of genes involved in fatty acid synthesis, triglyceride synthesis and lipid droplet formation [55], possibly resulting in greater FASN protein levels in SOL-EXO. Therefore, we hypothesized that skeletal muscle fibers are involved in intramuscular adipocyte adipogenesis mediated via myogenic exosomes as part of muscle-fat tissue interactions. Interestingly, fast and slow myogenic exosomes had the opposite effect on IMF deposition, suggesting that cells package specific proteins in exosomes to provide signal specificity and targeted delivery.

## Conclusions

In conclusion, we first isolated and identified porcine SOL-EXO and EDL-EXO, both of which had bilayer membrane disc-shaped morphologies and were approximately 84 nm in size. Furthermore, among the proteins differentially expressed between porcine SOL-EXO and EDL-EXO, FASN, GPRC5B, MINK1, PIN1, CCN2 and SUMO1 were among the top 10 upregulated or downregulated proteins and were implicated in adipogenic regulation partially through exosome transport. Importantly, SOL-EXO promoted lipid accumulation in LD intramuscular adipocytes, but EDL-EXO inhibited lipid accumulation in vitro. These findings provide novel insights into fast and slow myogenic exosome-mediated muscle-fat tissue crosstalk.

### Supplementary Information

**Additional file 1:** **Table S1. **The top 10 upregulating proteins between SOL-EXO vs. EDL-EXO. **Table S2.** The top 10 downregulating proteins between SOL-EXO vs. EDL-EXO.

**Additional file 2:** **Fig. S1.** Porcine SOL and EDL intramuscular preadipocytes were during proliferation and differentiation. **a** PorcineSOL and EDL on 3 days of age. **b** SOL and EDL preadipocytes during proliferation and differentiation. **Fig. S2.** Analysis of molecular weight and location of porcine SOL-EXO and EDL-EXO proteins. **a** Molecular weight of porcine SOL-EXO and EDL-EXO proteins. **b** Location of porcine SOL-EXO and EDL-EXO proteins. **Fig. S3.** Theexosomes of proliferation and differentiation C2C12 cells inhibited adipogensis in 3T3-L1 preadipocytes. **a** Experimental procedure. **b** TG content. **c** The protein levels of key lipogenic and lipolytic genes. Different lowercase letters indicate significant differences (*P* < 0.05).

## Abbreviations

ATGL: Adipose triglyceride lipase
CCN2: Cellular communication network factor 2
CEBPβ: CCATT enhancer-binding protein β
DAPI: 4′,6-diamidino-2-phenylindole
DMEM: Dulbecco's Modified Eagle Medium
EDL: Extensor digitorum longus
EDL-EXO: Extensor digitorum longus-exosomes
FABP4: Fatty acid binding protein 4
FASN: Fatty acid synthase
FBS: Fetal bovine serum
GDF5: Growth differentiation factor 5
GO: Gene Ontology
GAS: Gastrocnemius
HSL: Hormone-sensitive lipase
IBMX: 3-isobutyl-1-methylxanthine
IMF: Intramuscular fat
KEGG: Kyoto Encyclopedia of Genes and Genomes
LD: Longissimus dorsi
MINK1: Misshapen like kinase 1
MyoD: Myogenic differentiation 1
ORO: Oil Red O
PAX7: Paired box 7
PBS: Phosphate buffer saline
PIN1: Peptidyl-prolyl isomerase 1
PPARγ: Peroxisome proliferator–activated receptor-γ
RPMI: Roswell Park Memorial Institute
SEM: Scanning electronic microscopy
SOL: Soleus
SOL-EXO: Soleus-exosomes
SUMO1: Small ubiquitin-like modifier 1
TA: Tibialis anterior
TAZ: Transcriptional coactivator with PDZ-binding motif
TEM: Transmission electron microscope
TG: Triglyceride

## References

[1] Zhang Y, Zhang Y, Li H, Guo T, Jia J, Zhang P (2022). Comparison of nutrition and flavor characteristics of five breeds of pork in China. Foods.

[2] Chen L, Li J, Wang Y (2021). Current situation, cause analysis and countermeasures of China’s hog related products trade. Pract Foreign Econ Relat Trade.

[3] Webb EC, O'Neill HA (2008). The animal fat paradox and meat quality. Meat Sci.

[4] Gu H, Zhou Y, Yang J, Li J, Peng Y, Zhang X (2021). Targeted overexpression of PPARγ in skeletal muscle by random insertion and CRISPR/Cas9 transgenic pig cloning enhances oxidative fiber formation and intramuscular fat deposition. FASEB J.

[5] Essén-Gustavsson B, Karlsson A, Lundström K, Enfält AC (1994). Intramuscular fat and muscle fibre lipid contents in halothane-gene-free pigs fed high or low protein diets and its relation to meat quality. Meat Sci.

[6] Chen FF, Wang YQ, Tang GR, Liu SG, Cai R, Gao Y (2019). Differences between porcine longissimus thoracis and semitendinosus intramuscular fat content and the regulation of their preadipocytes during adipogenic differentiation. Meat Sci.

[7] Ren ZQ, Wang CY, Kou ZY, Cai R, Yang GS, Pang WJ (2023). In vivo estimation of lean percentage, fat percentage, and intramuscular fat content of boars by computed tomography. Scientia Agricultura Sinica.

[8] Joo ST, Kim GD, Hwang YH, Ryu YC (2013). Control of fresh meat quality through manipulation of muscle fiber characteristics. Meat Sci.

[9] Wosczyna MN, Perez Carbajal EE, Wagner MW, Paredes S, Konishi CT, Liu L (2021). Targeting microRNA-mediated gene repression limits adipogenic conversion of skeletal muscle mesenchymal stromal cells. Cell Stem Cell.

[10] Kim GD, Jeong JY, Jung EY, Yang HS, Lim HT, Joo ST (2013). The influence of fiber size distribution of type IIB on carcass traits and meat quality in pigs. Meat Sci.

[11] Zhang L, Guo Y, Wang L, Liu X, Yan H, Gao H (2020). Genomic variants associated with the number and diameter of muscle fibers in pigs as revealed by a genome-wide association study. Animal.

[12] Cho IC, Park HB, Ahn JS, Han SH, Lee JB, Lim HT (2019). A functional regulatory variant of MYH3 influences muscle fiber-type composition and intramuscular fat content in pigs. PLoS Genet.

[13] Li YH, Li FN, Duan YH, Guo QP, Wen CY, Wang WL (2018). Low-protein diet improves meat quality of growing and finishing pigs through changing lipid metabolism, fiber characteristics, and free amino acid profile of the muscle. J Anim Sci.

[14] Trajkovic K, Hsu C, Chiantia S, Rajendran L, Wenzel D, Wieland F (2008). Ceramide triggers budding of exosome vesicles into multivesicular endosomes. Science.

[15] Guescini M, Maggio S, Ceccaroli P, Battistelli M, Annibalini G, Piccoli G (2017). Extracellular vesicles released by oxidatively injured or intact C2C12 myotubes promote distinct responses converging toward myogenesis. Int J Mol Sci.

[16] Li S (2021). The basic characteristics of extracellular vesicles and their potential application in bone sarcomas. J Nanobiotechnol.

[17] Zhao R, Zhao T, He Z, Cai R, Pang W (2021). Composition, isolation, identification and function of adipose tissue-derived exosomes. Adipocyte.

[18] Zhou X, Liu Y, Zhang L, Kong X, Li F (2021). Serine-to-glycine ratios in low-protein diets regulate intramuscular fat by affecting lipid metabolism and myofiber type transition in the skeletal muscle of growing-finishing pigs. Anim Nutr.

[19] Guo L, Quan M, Pang W, Yin Y, Li F (2023). Cytokines and exosomal miRNAs in skeletal muscle-adipose crosstalk. Trends Endocrinol Metab.

[20] Wang S, Sun Y, Ren R, Xie J, Tian X, Zhao S (2019). H3K27me3 depletion during differentiation promotes myogenic transcription in porcine satellite cells. Genes (Basel).

[21] Li M, Liu Q, Xie S, Fu C, Li J, Tian C (2023). LncRNA TCONS_00323213 promotes myogenic differentiation by interacting with PKNOX2 to upregulate MyoG in porcine satellite cells. Int J Mol Sci.

[22] Wang T, Xu YQ, Yuan YX, Xu PW, Zhang C, Li F, et al. Succinate induces skeletal muscle fiber remodeling via SUNCR1 signaling. EMBO Rep. 2019;20(9):e47892. 10.15252/embr.201947892. 10.15252/embr.201947892 PMC6727026 31318145

[23] Supakankul P, Mekchay S (2016). Association of NLK polymorphisms with intramuscular fat content and fatty acid composition traits in pigs. Meat Sci.

[24] Chen FF, Xiong Y, Peng Y, Gao Y, Qin J, Chu GY (2017). miR-425-5p inhibits differentiation and proliferation in porcine intramuscular preadipocytes. Int J Mol Sci.

[25] Sun YM, Qin J, Liu SG, Cai R, Chen XC, Wang XM, et al. *PDGFRα* regulated by miR-34a and *FoxO1* promotes adipogenesis in porcine intramuscular preadipocytes through Erk signaling pathway. Int J Mol Sci. 2017;18(11):2424. 10.3390/ijms18112424. 10.3390/ijms18112424 PMC5713392 29140299

[26] Pang WJ, Xiong Y, Wang Y, Tong Q, Yang GS (2013). Sirt1 attenuates camptothecin-induced apoptosis through caspase-3 pathway in porcine preadipocytes. Exp Cell Res.

[27] Gualerzi A, Niada S, Giannasi C, Picciolini S, Morasso C, Vanna R (2017). Raman spectroscopy uncovers biochemical tissue-related features of extracellular vesicles from mesenchymal stromal cells. Sci Rep.

[28] Wang ZG, He ZY, Liang S, Yang Q, Cheng P, Chen AM (2020). Comprehensive proteomic analysis of exosomes derived from human bone marrow, adipose tissue, and umbilical cord mesenchymal stem cells. Stem Cell Res Ther.

[29] Joo ST, Joo SH, Hwang YH (2017). The relationships between muscle fiber characteristics, intramuscular fat content, and fatty acid compositions in *M. longissimus lumborum* of Hanwoo Steers. Korean J Food Sci Anim Resour.

[30] Cai L, Huang Y, Zhong L, Zou X, Ji J, Liu X (2023). Using phenotypic and genotypic big data to investigate the effect of muscle fiber characteristics on meat quality and eating quality traits in pigs. Meat Sci.

[31] Xu Z, You W, Chen W, Zhou Y, Nong Q, Valencak TG (2021). Single-cell RNA sequencing and lipidomics reveal cell and lipid dynamics of fat infiltration in skeletal muscle. J Cachexia Sarcopenia Muscle.

[32] Li X, Fu X, Yang G, Du M (2020). Review: Enhancing intramuscular fat development via targeting fibro-adipogenic progenitor cells in meat animals. Animal.

[33] Wang L, Gao P, Li C, Liu Q, Yao Z, Li Y (2023). A single-cell atlas of bovine skeletal muscle reveals mechanisms regulating intramuscular adipogenesis and fibrogenesis. J Cachexia Sarcopenia Muscle.

[34] Kopinke D, Roberson EC, Reiter JF (2017). Ciliary hedgehog signaling restricts injury-induced adipogenesis. Cell.

[35] Kalluri R, LeBleu VS (2020). The biology, function, and biomedical applications of exosomes. Science.

[36] Aoi W, Tanimura Y (2021). Roles of skeletal muscle-derived exosomes in organ metabolic and immunological communication. Front Endocrinol (Lausanne).

[37] Litviňuková M, Talavera-López C, Maatz H, Reichart D, Worth CL, Lindberg EL (2020). Cells of the adult human heart. Nature.

[38] Qin M, Xing L, Wu J, Wen S, Luo J, Chen T, et al. Skeletal muscle-derived exosomal miR-146a-5p inhibits adipogenesis by mediating muscle-fat axis and targeting GDF5-PPARγ signaling. Int J Mol Sci. 2023;24(5):4561. 10.3390/ijms24054561. 10.3390/ijms24054561 PMC10003660 36901991

[39] Li W, Wen S, Wu J, Zeng B, Chen T, Luo J (2021). Comparative analysis of microRNA mxpression profiles between skeletal muscle and adipose-derived exosomes in pig. Front Genet.

[40] Arya SB, Collie SP, Parent CA. The ins-and-outs of exosome biogenesis, secretion, and internalization. Trends Cell Biol. 2023;34(2):90–108. 10.1016/j.tcb.2023.06.006. 10.1016/j.tcb.2023.06.006 PMC10811273 37507251

[41] Romancino DP, Paterniti G, Campos Y, De Luca A, Di Felice V, d'Azzo A (2013). Identification and characterization of the nano-sized vesicles released by muscle cells. FEBS Lett.

[42] Lenassi M, Cagney G, Liao M, Vaupotic T, Bartholomeeusen K, Cheng Y (2010). HIV Nef is secreted in exosomes and triggers apoptosis in bystander CD4^+^ T cells. Traffic.

[43] Rowland LA, Guilherme A, Henriques F, DiMarzio C, Munroe S, Wetoska N (2023). De novo lipogenesis fuels adipocyte autophagosome and lysosome membrane dynamics. Nat Commun.

[44] Kim YJ, Greimel P, Hirabayashi Y (2018). GPRC5B-mediated sphingomyelin synthase 2 phosphorylation plays a critical role in insulin resistance. iScience.

[45] Hyodo T, Ito S, Hasegawa H, Asano E, Maeda M, Urano T (2012). Misshapen-like kinase 1 (MINK1) is a novel component of striatin-interacting phosphatase and kinase (STRIPAK) and is required for the completion of cytokinesis. J Biol Chem.

[46] Song WW, McLennan SV, Tam C, Williams PF, Baxter RC, Twigg SM (2015). CCN2 requires TGF-β signalling to regulate CCAAT/enhancer binding proteins and inhibit fat cell differentiation. J Cell Commun Signal.

[47] Pazienza V, Panebianco C, Rappa F, Memoli D, Borghesan M, Cannito S, ey al. Histone macroH2A1.2 promotes metabolic health and leanness by inhibiting adipogenesis. Epigenetics Chromatin. 2016;9:45. 10.1186/s13072-016-0098-9. 10.1186/s13072-016-0098-9 PMC5078890 27800025

[48] Han Y, Lee SH, Bahn M, Yeo CY, Lee KY (2016). Pin1 enhances adipocyte differentiation by positively regulating the transcriptional activity of PPARγ. Mol Cell Endocrinol.

[49] Liu B, Wang T, Mei W, Li D, Cai R, Zuo Y (2014). Small ubiquitin-like modifier (SUMO) protein-specific protease 1 de-SUMOylates Sharp-1 protein and controls adipocyte differentiation. J Biol Chem.

[50] Anghel SI, Wahli W (2007). Fat poetry: a kingdom for PPAR gamma. Cell Res.

[51] Zhu Q, Wang D, Liang F, Tong X, Liang Z, Wang X (2022). Protein arginine methyltransferase PRMT1 promotes adipogenesis by modulating transcription factors C/EBPβ and PPARγ. J Biol Chem.

[52] Tang QQ, Lane MD (2012). Adipogenesis: from stem cell to adipocyte. Annu Rev Biochem.

[53] Morak M, Schmidinger H, Riesenhuber G, Rechberger GN, Kollroser M, Haemmerle G (2012). Adipose triglyceride lipase (ATGL) and hormone-sensitive lipase (HSL) deficiencies affect expression of lipolytic activities in mouse adipose tissues. Mol Cell Proteomics.

[54] Guo YY, Li BY, Xiao G, Liu Y, Guo L, Tang QQ (2022). Cdo1 promotes PPARγ-mediated adipose tissue lipolysis in male mice. Nat Metab.

[55] Komiya Y, Sawano S, Mashima D, Ichitsubo R, Nakamura M, Tatsumi R (2017). Mouse soleus (slow) muscle shows greater intramyocellular lipid droplet accumulation than EDL (fast) muscle: fiber type-specific analysis. J Muscle Res Cell Motil.

