# Supplementary material for: Identification of porcine fast/slow myogenic exosomes and their regulatory effects on lipid accumulation in intramuscular adipocytes
Source: J Anim Sci Biotechnol. 2024 Jun 2;15:73. doi: 10.1186/s40104-024-01029-0 (PMC11144342; doi:10.1186/s40104-024-01029-0)
Supplement: Supplementary file 1 — Additional file 1: Table S1. The top 10 upregulating proteins between SOL-EXO vs. EDL-EXO. Table S2. The top 10 downregulating proteins between SOL-EXO vs. EDL-EXO. [file 40104_2024_1029_MOESM1_ESM.docx]

**Table S1** The top 10 upregulating proteins between SOL-EXO vs. EDL-EXO differential expression

| **Protein name** | **fd** | **log_2_fc** | **qval** |
| --- | --- | --- | --- |
| MINK1 | 4.21933 | 2.077015 | 0.024548 |
| CLIC2 | 2.547012 | 1.348806 | 0.177476 |
| PN-1 | 2.046607 | 1.033234 | 0.008625 |
| FASN | 1.922431 | 0.942932 | 0.000827 |
| GPRC5B | 1.921878 | 0.942517 | 0.049727 |
| CDH2 | 1.768281 | 0.822348 | 0.019787 |
| PSMB7 | 1.532708 | 0.616083 | 0.026202 |
| NRP1 | 1.529003 | 0.612591 | 0.004583 |
| ALDOA | 1.287097 | 0.364121 | 0.020895 |
| LDHA | 1.218845 | 0.285525 | 0.000836 |

**Table S2** The top 10 downregulating proteins between SOL-EXO vs. EDL-EXO differential expression

| **Protein name** | **fd** | **log_2_fc** | **qval** |
| --- | --- | --- | --- |
| DCTN6 | 0.369928 | -1.43468 | 0.049012 |
| API5 | 0.403586 | -1.30905 | 0.004177 |
| FXYD6 | 0.468187 | -1.09487 | 0.036387 |
| DBT | 0.608965 | -0.71557 | 0.026184 |
| CCN2 | 0.626313 | -0.67504 | 0.00424 |
| PIN1 | 0.666139 | -0.58611 | 0.024704 |
| PFKFB2 | 0.713689 | -0.48663 | 0.031613 |
| APOH | 0.735853 | -0.44251 | 0.024654 |
| METTL7A | 0.742701 | -0.42915 | 0.007819 |
| SUMO1 | 0.748900 | -0.41715 | 0.008979 |
