# Supplementary figures and images for: Identification of porcine fast/slow myogenic exosomes and their regulatory effects on lipid accumulation in intramuscular adipocytes
Source: J Anim Sci Biotechnol. 2024 Jun 2;15:73. doi: 10.1186/s40104-024-01029-0 (PMC11144342; doi:10.1186/s40104-024-01029-0)

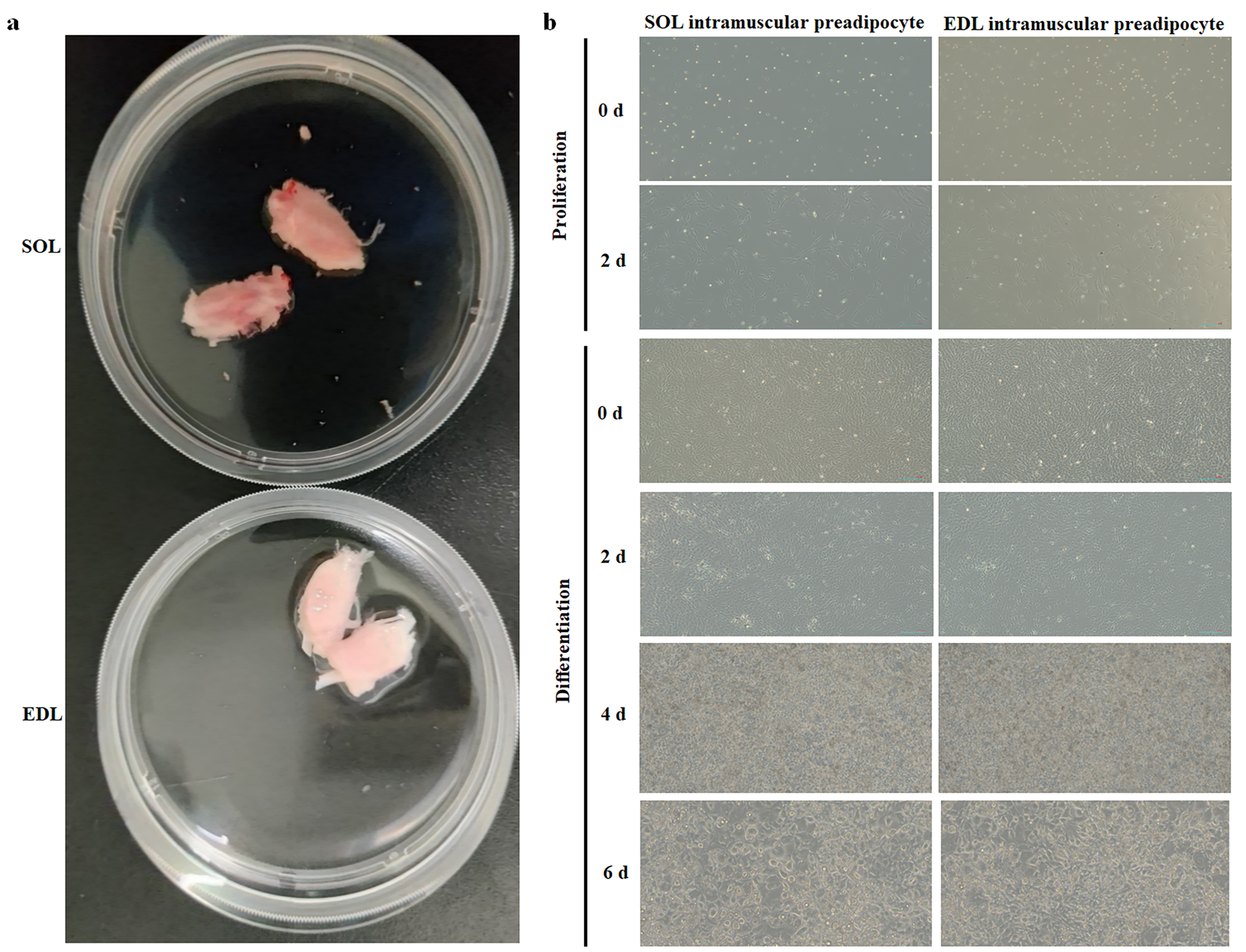

Supplement: Supplementary file 2 — Additional file 2: Fig. S1. Porcine SOL and EDL intramuscular preadipocytes were during proliferation and differentiation. a PorcineSOL and EDL on 3 days of age. b SOL and EDL preadipocytes during proliferation and differentiation. Fig. S2. Analysis of molecular weight and location of porcine SOL-EXO and EDL-EXO proteins. a Molecular weight of porcine SOL-EXO and EDL-EXO proteins. b Location of porcine SOL-EXO and EDL-EXO proteins. Fig. S3. Theexosomes of proliferation and differentiation C2C12 cells inhibited adipogensis in 3T3-L1 preadipocytes. a Experimental procedure. b TG content. c The protein levels of key lipogenic and lipolytic genes. Different lowercase letters indicate significant differences (P < 0.05). [file 40104_2024_1029_MOESM2_ESM.zip › Fig. S1.tif]

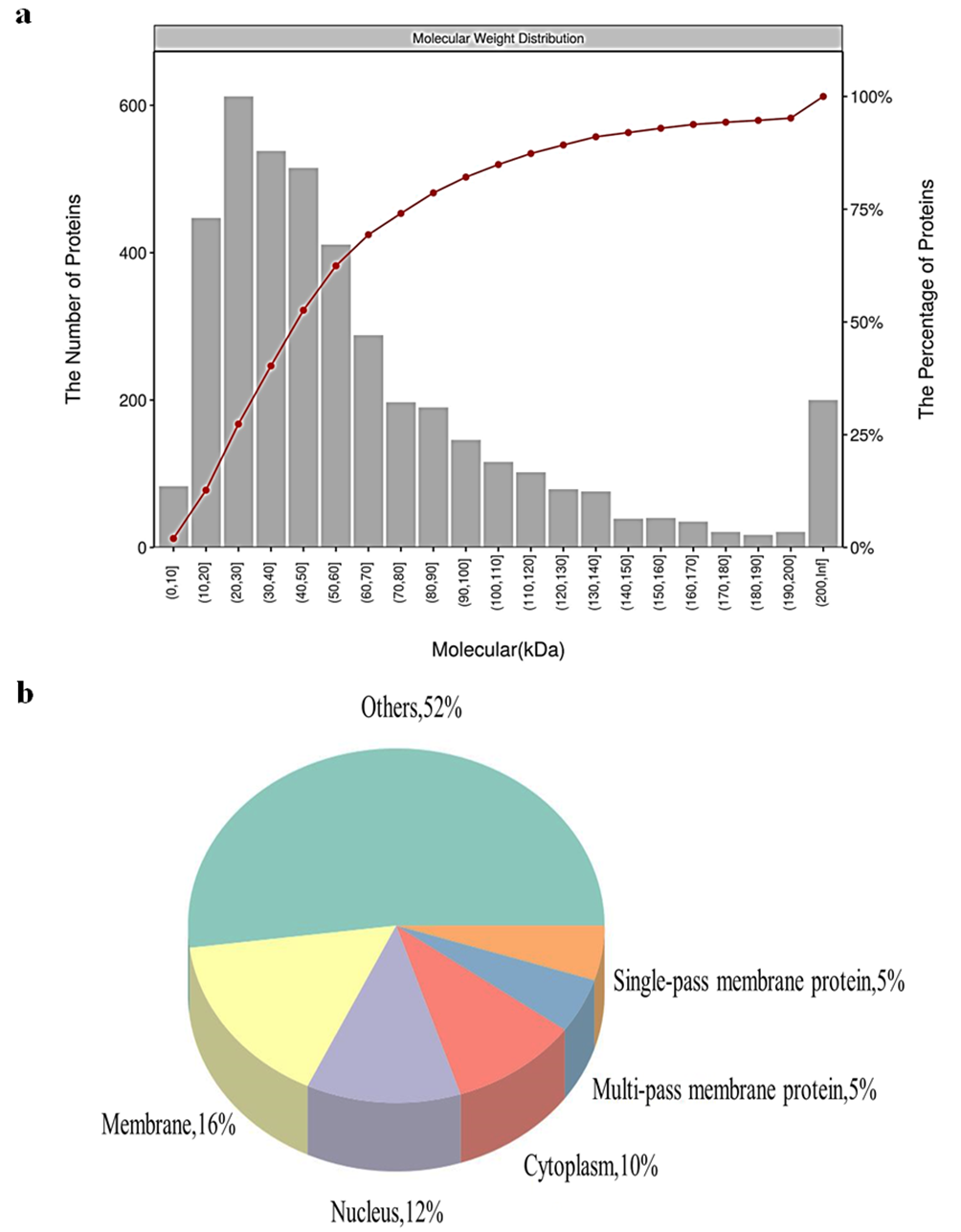

Supplement: Supplementary file 2 — Additional file 2: Fig. S1. Porcine SOL and EDL intramuscular preadipocytes were during proliferation and differentiation. a PorcineSOL and EDL on 3 days of age. b SOL and EDL preadipocytes during proliferation and differentiation. Fig. S2. Analysis of molecular weight and location of porcine SOL-EXO and EDL-EXO proteins. a Molecular weight of porcine SOL-EXO and EDL-EXO proteins. b Location of porcine SOL-EXO and EDL-EXO proteins. Fig. S3. Theexosomes of proliferation and differentiation C2C12 cells inhibited adipogensis in 3T3-L1 preadipocytes. a Experimental procedure. b TG content. c The protein levels of key lipogenic and lipolytic genes. Different lowercase letters indicate significant differences (P < 0.05). [file 40104_2024_1029_MOESM2_ESM.zip › Fig. S2.tif]

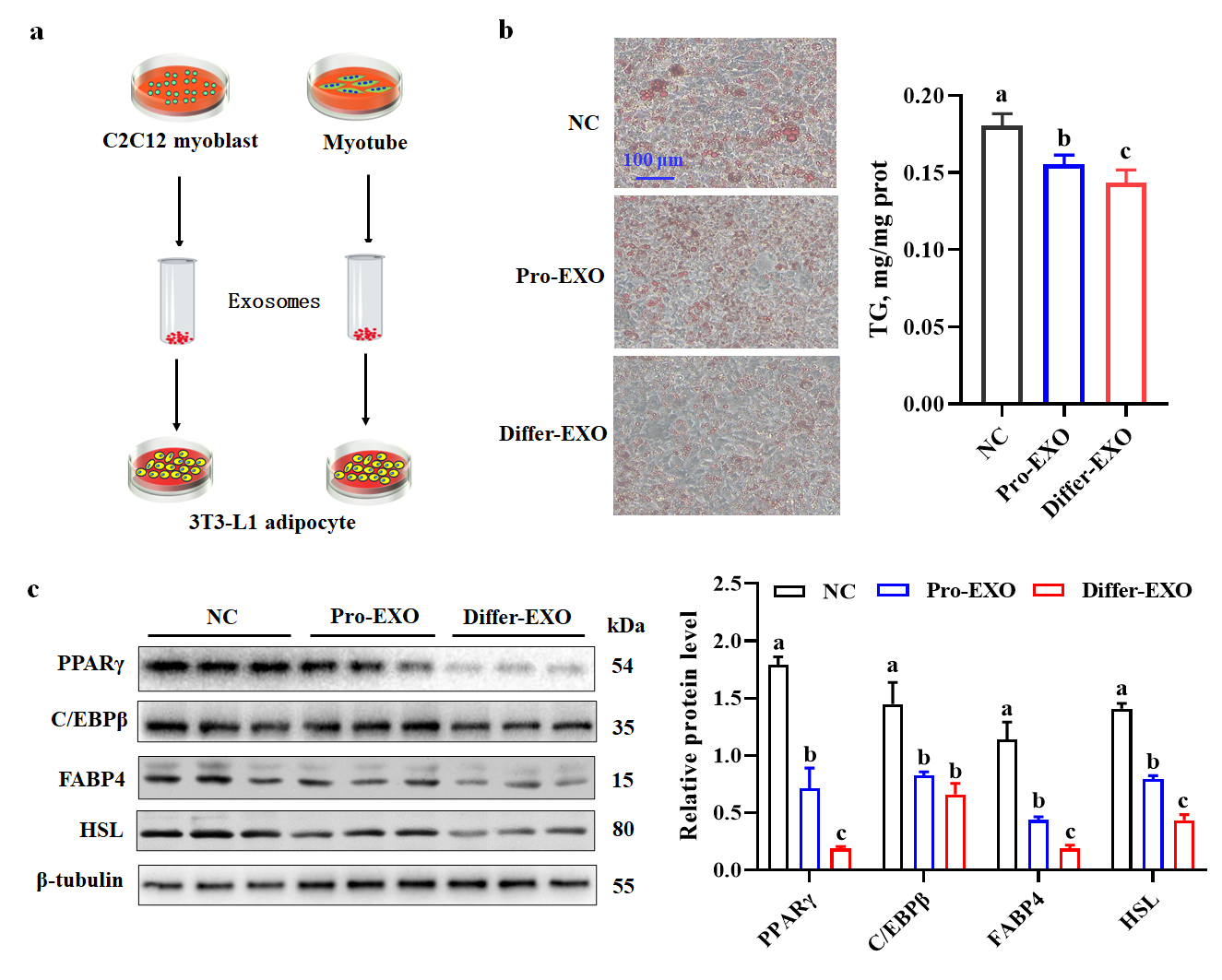

Supplement: Supplementary file 2 — Additional file 2: Fig. S1. Porcine SOL and EDL intramuscular preadipocytes were during proliferation and differentiation. a PorcineSOL and EDL on 3 days of age. b SOL and EDL preadipocytes during proliferation and differentiation. Fig. S2. Analysis of molecular weight and location of porcine SOL-EXO and EDL-EXO proteins. a Molecular weight of porcine SOL-EXO and EDL-EXO proteins. b Location of porcine SOL-EXO and EDL-EXO proteins. Fig. S3. Theexosomes of proliferation and differentiation C2C12 cells inhibited adipogensis in 3T3-L1 preadipocytes. a Experimental procedure. b TG content. c The protein levels of key lipogenic and lipolytic genes. Different lowercase letters indicate significant differences (P < 0.05). [file 40104_2024_1029_MOESM2_ESM.zip › Fig. S3.tif]
